# Meeting Review: Bioinformatics of Biochips: Accelerating Discovery in Functional Genomics

**DOI:** 10.1002/cfg.179

**Published:** 2002-08

**Authors:** Douglas Roy, Thorsten Forster, Sean McGeever, Kevin Robertson, Peter Ghazal

**Affiliations:** 1 Scottish Centre for Genomic Technology and Informatics University of Edinburgh UK; 2 SCGTI Summerhall Edinburgh EH91QH UK


**Comparative and Functional Genomics**
*Comp Funct Genom* 2002; **3**: 380–386.
Published online in Wiley InterScience (www.interscience.wiley.com). **DOI:** 10.1002/cfg.179

**Feature**

### Meeting Review: Bioinformatics of Biochips: Accelerating Discovery in Functional Genomics

The Royal Society of Edinburgh, George Street, Edinburgh, UK, 20 March 2002

Douglas Roy, Thorsten Forster, Sean McGeever, Kevin Robertson and Peter Ghazal*
*Scottish Centre for Genomic Technology and Informatics, University of Edinburgh, UK*

*Correspondence to:
Peter Ghazal, SCGTI,
Summerhall, Edinburgh
EH9 1QH, UK.
E-mail: p.ghazal@ed.ac.uk*

Received: 29 May 2002
Accepted: 6 June 2002

### Introduction

The unprecedented scale and content of genomic and proteomic information now emerging from global sequencing and analysis efforts offer new opportunities in biological research. Technological and computational developments have enabled first phase analytical platforms for genomic and proteomic studies. However, the ultimate goal will be to integrate data from diverse analytical platforms, so that biological systems can be modelled with increasing complexity to achieve understanding at the systems level. The new techniques of microarray technology and high-throughput screening (HTS) proteomics are already providing new insights into cell form and function. These approaches are poised to revolutionize much of biological research methodology, but will require the increasing fusion of biological sciences with mathematics, computing and physical sciences to generate platform technologies and approaches for the specific and HTS analysis of biological processes. This workshop was held to review the central role of bioarrays in genomic and proteomic studies and to examine their potential for future applications. It was also critical to consider the requirement for bioinformatic and computational tools and methods to enable rational handling and interpretation of data.

This event was organized as a research workshop under the auspices of the Royal Society of Edinburgh and the Wellcome Trust. The report below considers the three major bioarray-related themes resulting from the meeting: analytical methods; functional genomic applications and the development of new technologies. These key areas will require fusion to provide a fuller understanding of biological systems (Figure 1).

### Analytical methods: the statistics behind bioarray analysis

Claus Mayer (Rowett Research Institute, Aberdeen, and BIOSS, Edinburgh, UK) emphasized the role of statisticians in microarray technology and research. He pointed out that cooperation between statisticians and biologists is useful and profitable for both sides. Although the number of purely statistical papers on microarray technology has grown exponentially, they are still in a minority compared to microarray-related biology papers.

Copyright © 2002 John Wiley & Sons, Ltd.

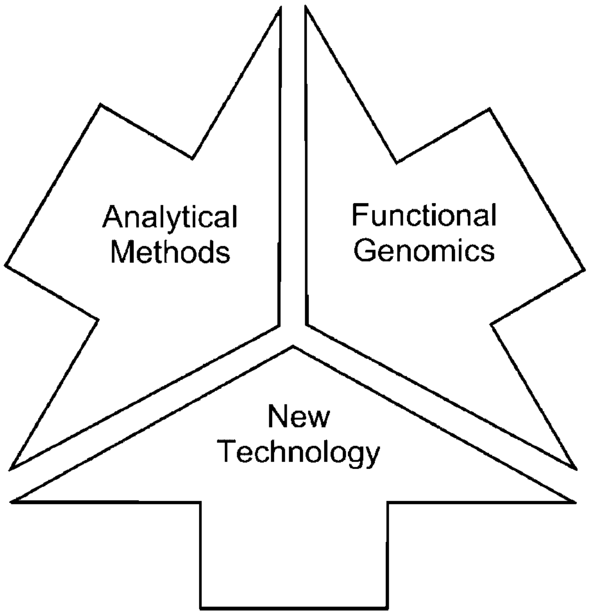

**Figure 1.** Fusions required for systems biology

This indicates, however, that statistical methods would continue to impact in microarray research. Statisticians should make use of the opportunity to develop ideas and models for analysing images and data structures (amount of data, dimensionality, sources of noise and variation) that are different from 'traditional' approaches. Despite this complexity, the actual background and implications behind microarray technology are still easier to understand than most other areas of biology. Biologists should make use of the opportunity to apply structured analysis methods and approaches to microarray data, resulting in greater confidence in their numerical results. This in turn will produce a better foundation for biological interpretations. He listed the areas in which statistics can be utilized in microarray research: experimental design; image processing; transformation/normalization; and single gene and multivariate analysis. In view of this, it is beneficial to involve statisticians early on in the planning of a microarray project. In terms of practical recommendations for the processing of microarray data, he outlined the following:

- Estimation of systematic background trends might be preferable to subtracting individual backgrounds.
- Replication is required at all experimental levels.
- Log-transformations to be carried out on ratio data and absolute values, or alternatively, gamma or arcinh transformations can be applied.
- Normalization methods need to be decided on a per-experiment basis.
- Investigation of distributions and histograms should be part of the analysis process.

Concluding, he pointed out that statistical methods should be integrated with bioinformatics and other technologies and approaches for the complete analysis of microarray data.

John Quackenbush (Institute for Genomic Research, TIGR, Rockville, USA) discussed practical approaches in the analysis of microarray data. He pointed to the important relationship between study design and data normalization and analysis. The collation of as much information as possible about the experiment is required. Ideally, this information would adhere to the MIAME standards discussed by Brazma (below). He compared a standard microarray reference design to the loop-design concept and pointed out their advantages and disadvantages. Reference design is simple, robust and easily extensible, but provides limited data for experimental samples. Loop designs provide better statistics and direct cross-sample comparisons, but they demand a larger amount of biological material for analysis and are not as robust. Overall, loop designs are preferable over reference designs if properly formulated. Basic experimental design should include independent biological replicates incorporating self-to-self comparisons or 'dye-swap' experiments. A number of approaches for dye-effect normalization were put forward. The essential requirement is to perform normalization or a combination of normalization methods initially; and that the particular method should be decided on a per-experiment basis. Whether global or gene subset normalization is employed depends on the experimental design and array layout. As a useful method of visualizing the relationship between dye-effect and the magnitude of expression measurements he suggested the R–I plot [$\log_2$(R/G) vs. $\log_{10}$(R*G)], which is closely related to the M–A plot suggested by Terry Speed's group (UCLA, Berkeley, USA).

An explanation of the ideas behind various types of cluster analysis to measure the similarity of expression profiles for genes was given. No general recommendations of the best parameter

Copyright © 2002 John Wiley & Sons, Ltd. 

choices or clustering approach can yet be made. This is because all types and methods have their own features and highlight different aspects of the underlying data. Concluding, he pointed out that, apart from microarray technology, there is a wealth of information emerging from all genomic and related technologies. The challenge is to combine these data sets to gain a deeper insight into biological processes. However, it was pointed out that additional layers of data demand increasing caution when inferring biological significance from statistical significance. The role of the biologist will continue to be essential in all stages of design, analysis and interpretation. During the course of his talk, some of the computational and bioinformatic tools TIGR (**http://www.tigr.org/software/**) and others have developed were presented:

- GENE INDEX (integration of several databases, EST data including a variety of tools for orthologue and paralogue mapping).
- RESOURCERER (annotation tool and resource comparison based on TIGR Gene Index).
- MEV (multi experiment viewer, data normalization, analysis and visualization tool).
- MADAM (microarray data manager, under development).
- MIDAS (microarray information and data analysis suite, under development).
- ANOVA (statistical tools, TIGR/Jackson Laboratories).
- MAD (downloadable database of in-house microarray experiments on mouse genome, Jackson Laboratory).

Andy Brass (University of Manchester, UK) presented ideas for the use of gene expression distribution on a per-chip basis. In contrast to examining expression of individual genes across conditions or time-points, this approach concentrates on global gene expression across entire arrays or genomes. He identified four features that, if monitored, could be used for quality control and data normalization:

- Microarray data usually demonstrate log-normal distribution.
- Microarray data fit Benford's law fairly well, i.e. one can expect 1 to be the first logscale digit of an intensity value in about 30% of cases, 2 will come up in about 18% of cases, 3 in 12%, etc, so that $[P(D) = \log_{10}(1 + D^{-1})]$.
- Microarray data also appear to fit Zipf's law, which states that the quantity under study is inversely proportional to the rank, i.e. proportional to 1, 1/2, 1/3, etc. Therefore, if using logs of value and rank, if the highest expression (rank 1) is 10, then the second highest expression is 10−log(2), third highest 10−log(3), etc.
- Microarray data appear to show a relationship between width of distribution and size of genome under study.

Knowing what characteristics the data distribution on an array conform to might facilitate quality control and normalization by comparing and adjusting the actual distribution to these rules. It is possible that the biological significance of these phenomena is related to the average number of transcription factor binding sites per gene. In summary, it was proposed that signal intensity distributions for an array are based on real biology, and that biology as well as data can be successfully modelled by log-normal distributions. This could prove to be the basis for useful QC and normalization tools. Options for this type of data processing will be included in a new release of the MaxD microarray data warehouse and visualization environment.

Alvis Brazma (EMBL-EBI, Cambridge, UK) presented a discussion of microarray data standards, databases and data mining, which explored some of the challenges inherent in both the biological and computational complexity of microarray experiments. Setting the scene, he first described the various elements and operations that together comprise microarray experiments. The scale and complexity of the data required to describe and record such experiments, the protocols used in them and the expression data they produce raise questions about how to assess the quality and relevance of data for the interpretation of experimental results. One approach is to implement standardizations of data capture and analysis as an approach to reduce the scale and complexity of data recorded for each microarray experiment. However, standardization is challenging and requires consistent and coherent procedures for gene annotation, consistent data exchange formats and continuity of experimental measurements. To meet these challenges, the Microarray Gene Expression Data (MGED) group has been introduced to establish consensus and common practice in the areas of information content, data exchange formats,

Copyright © 2002 John Wiley & Sons, Ltd.

biological sample ontologies and data normalization strategies. The first of these standardization efforts, the Minimum Information About a Microarray Experiment (MIAME), was introduced and the scope of its application, including sample identification, extraction, labelling and hybridization, was outlined. Description was given of the publicly available MIAMExpress and ArrayExpress tools as reference implementations of the MIAME standardization effort. Finally, a description of Expression Profiler and other computational tools for the analysis, visualization and interpretation of microarray gene expression data was given (for applications described, see: (**http://www.ebi.ac.uk/microarray/index.html**)

### From genes to function

Dr Phil Butcher (SGHMS, London, UK) described the multi-user bacterial pathogen array facility based at St. George's Medical School, London (**http://www.sghms.ac.uk/depts/medmicro/bugs/bugs_content_frame.htm**). This is a Wellcome Trust-funded initiative to provide DNA microarrays for a range of pathogenic bacteria. Functional genomics requires the fusion of different analytical platforms, such as genomics, proteomics and metabolomics, for the full analysis of complex biological systems, e.g. the interplay between host and pathogen during bacterial infection. The primary aim of the unit at St. George's is the production of DNA microarrays for several pathogenic bacteria. Arrays are fabricated using specific short PCR probes generated using specifically designed primers to bacterial gene sequences. The main focus of the talk was the application of genomic and proteomic technologies in the analysis of *Mycobacterium paratuberculosis* physiology. *M. paratuberculosis* is the causative agent of tuberculosis (TB), the worldwide incidence of which continues to increase. The development of an array platform for the genome of this organism was described. The array has been useful for comparative genotyping of *M. paratuberculosis* strains and mutants, and this HTS genotyping approach will be generally applicable to other bacterial pathogens.

Examples of RNA transcriptional profiling experiments using the array were also given. However, there are significant experimental problems associated with the analysis of bacterial RNA. For instance, 98% of bacterial RNA is ribosomal, leading to problems in generating sufficient yields of messenger RNA. Bacterial RNA is notoriously labile (average half-life typically less than 2 min) and very prone to degradation during extraction. In addition, bacterial transcripts are extremely tightly regulated, and there are very rapid switches in transcription profiles in response to changing environmental or physiological conditions. Bacterial messenger RNA is not polyadenylated, so gene-specific priming is usually employed during the target labelling step. Lysis of bacteria can be problematic and time consuming and may generate contaminants, which must be subsequently removed. These problems will require improvements in experimental methods for sample preparation and labelling. Nevertheless, examples were given of the microarray gene expression profile of *M. paratuberculosis* in response to stress conditions *in vitro* and during infection *in vivo*. A range of similar problems is encountered in proteomic analysis involving sampling and extraction of bacterial proteins for 2D gel separation and mass spectroscopic analysis. In both genomic and proteomic studies, very consistent methods of experimentation, including rapid processing, must be developed and adhered to.

Keith Vass (CRC Beatson Institute, Glasgow, UK) described the microarray-related research under way at the Beatson Institute. Significant research activity is concentrated on collaborative projects using the microarray platforms, experimental samples and approaches from the *M. paratuberculosis* research consortium described by Phil Butcher (see above). The need for appropriate tools for data normalization and visualization to analyse these experiments correctly was outlined. An ANOVA-based program has been developed in conjunction with the Department of Statistics at Glasgow University that accounts for the many sources of variation in microarray experiments and uses the background signal for the correction of spatial effects on the array. In order to classify operon usage during *M. paratuberculosis* growth profile experiments, analysis of co-variance and correlation maps of expression from adjacent genes were used. This approach should indicate functional relationships useful for the global mapping of operon activity.

Tom Freeman (HGMP-RC, Cambridge, UK) described the activities of the microarray unit at

Copyright © 2002 John Wiley & Sons, Ltd.

the MRC-HGMP resource centre based at Hinxton, Cambridge (**http://www.hgmp.mrc.ac.uk/Research/Microarray/index.jsp**). This unit has been set up by strategic funding from the MRC and other agencies to provide access to array technology for academic researchers within the UK. The unit provides training and support protocols for DNA microarray applications, and the design and fabrication of new array sets. The unit also provides access to the Affymetrix Gene Chip System. The remit of the unit is the dissemination of human and mouse arrays to the academic community. These will be free of charge, but prior application is required. The unit holds several large PCR-based probe sets, which are amplified using high-throughput robotics available at HGMP. These are arrayed onto glass slides, most usually via amino linkage. A number of control probes are added, including a range of spiking controls for genes considered suitable for microarray normalization. The quality of PCR libraries is of critical importance, and those used by HGMP are sequence-verified and well curated to reduce insert or phage contamination errors. However, the logistical problems associated with PCR based arrays were highlighted. The main problem is the consistent re-amplification of large clone sets to the required concentration and specificity. This process incurs considerable cost in terms of materials, time and personnel. There are other problems concerned with PCR arrays, including availability of probe sets and potential specificity.

Consequently, long oligonucleotide probes are finding increasing favour for the construction of DNA microarrays. Currently, oligos in the region of 50–70 mer are specifically designed to represent genes of interest. Although relatively expensive to procure as a one-off set, oligomer-based arrays can be produced with a high level of accuracy and consistency. A major benefit of oligomers is the specificity and accuracy of probe design, which will become increasingly sophisticated to generate exon-specific probes for multigene families and the detection of splice variants. The HGMP have access to a number of mammalian oligo probe sets, and genome-wide sets are under development. Also highlighted was the development of a database and LIMS architecture dedicated to the activities of the unit. Finally, Dr Freeman drew attention to a number of considerations important for successful array projects. These include the importance of experimental design and definition of the biological system under consideration. Experiments should include biological replicates and the quality of the data depends on the selection of consistent and high quality arrays to be used, in conjunction with effective and well-characterized experimental protocols. Data should be compared to the literature and verified by independent means where possible. The consistent management of data and the consideration of MIAME standards will be a prerequisite for publication.

Steve Kay (The Novartis Research Foundation, Scripps Institute and Phenomix, San Diego, USA) began his presentation by reviewing current technologies for biological analyses at a molecular, cellular and organismal level and proceeded to describe his work on the construction of an RNA expression atlas at the Novartis Foundation. The Atlas project has involved a programme of transcriptional profiling in 50 human and mouse tissues using the Affymetrix GeneChip platform. Professor Kay highlighted the reassuring discovery that, in general, organ-specific expression profiles are very similar in humans and mice. It is likely that the expression atlas will become an important resource in the future and data from this study will soon be available at (**http://expression.gnf.org**). Indeed, the atlas has already been used to derive the molecular signatures of many commonly fatal carcinomas.

Professor Kay proceeded to describe his academic work on circadian rhythms in nature, undertaken at The Scripps Research Institute. Global gene expression profiling studies have demonstrated that hundreds of genes are ‘clock’-regulated and cycle throughout a 24 h period in a variety of organisms. An example of such a gene is that which encodes the phenylpropanoid enzyme in plants. It was pointed out that three independent studies in this field, all attempting to identify clock-regulated genes, have only identified 16 genes in common. This indicates that even if a careful experimental design and a structured approach to data analysis are adopted, subtle differences in strategy may yield notably different answers. Examples of the tissue-specific control of clock gene expression were given, e.g. microarray experiments have demonstrated that the SCN master oscillator in the brain controls the ‘rhythm’ of gene expression in the liver.

Copyright © 2002 John Wiley & Sons, Ltd. 

Finally, there was description of a mouse forward genetics project carried out with the company Phenomix. This project has involved a genome-wide mutagenesis and screening program, which aims to identify genes involved in neurological, immunological and physiological processes and disease states. This screening process has exploited a novel high-throughput antibody array system for serum profiling. The antibody array has the capability to simultaneously measure up to 96 analytes in less than 20 μl serum and can be used to screen more than 200 mice per week. This technology has allowed great advances in parallel biological and mapping analyses.

Douglas Roy (SCGTI, Edinburgh, UK) reinforced that biology increasingly requires HTS experimental and analytical methods to accommodate the scale and complexity of genomic and proteomic information now available. The Scottish Centre for Genomic Technology and Informatics was introduced as an example of how a centralized approach incorporating genomic technologies and bioinformatics can address the requirements for functional genomic analysis. The SCGTI (**http://www.gti.ed.ac.uk**) operates as a major collaborative focus for the application of genomic technologies combined with statistical, bioinformatic and computational methods for data handling and visualization. The SCGTI collaborates on a wide range of projects covering mammalian systems, model organisms and pathogens. Several examples of the types of projects under way were outlined. For these projects, a variety of DNA and protein microarrays are fabricated in house, and the Centre also utilizes the Affymetrix Gene Chip system. There is close collaboration with groups wishing to apply microarray analysis to biological problems. A number of novel genomic and proteomic array platforms are under development, including the analysis of splice variants in yeast, genomic structural studies and the detection of blood typing proteins. The SCGTI is developing a relational database architecture based on the NCGR GeneX schema to accommodate the data analysis and LIMS requirements of collaborating research groups. A future aim of the Centre will be the incorporation and integration of diverse data sets (genomics, proteomics and imaging) to model biological systems in greater complexity.

### Emerging technologies

Jon Cooper (University of Glasgow, UK) discussed the impact of nanotechnology on drug discovery. An overview was given of the relative scale and manufacturing processes required for the production of field effect transistors. Discussion then followed of the advantages of nanotechnologies and how they might be applied to biomedical research. Description was given of micro-fluidic systems, which allow the highly controlled flow and mixing of minute quantities of liquids. An overview was given of his work on a micro-machined chip for DNA hybridization studies and the development of micro- and nano-sensors for the study of intracellular metabolic activity. In particular, he highlighted a system for the study of thermal measurements in single cardiomyocyte cells following exposure to a variety of environmental changes. Finally, recent studies undertaken in conjunction with the company Adaptive Screening were described. These studies have focused on the development of novel high-density cell-screening devices. Such devices can be used for ultra-high-throughput cell-based functional assays.

Tony Cass (Imperial College, London, UK) outlined the challenges facing the high-throughput analysis of protein interactions. It is predominantly proteins that impart form and function to living systems, and protein binding is the key biological event inherent to signalling and transmission. Human genomic analysis currently predicts 35 000 genes coding for around 150 000 proteins. At one level of analysis, the proteome can be considered as a matrix of 150 000 protein-binding sites. However, there are problems in the development of HTS proteomic platforms for protein interaction studies. Thus, far fewer than 1% of proteins are represented in protein databases, with new additions running at approximately 1000 per annum. Proteins are classified into subfamilies and no method exists for global protein interaction analysis similar to those used in the microarray analysis of nucleic acids. This poses a problem for pharmaceutical research where, despite an explosion of information about potential genetic targets and the generation of comprehensive compound libraries for analysis, there has not been a commensurate increase in lead compound discovery and target validation. Existing methods of compound validation are proving increasingly costly, due to the failure

Copyright © 2002 John Wiley & Sons, Ltd.

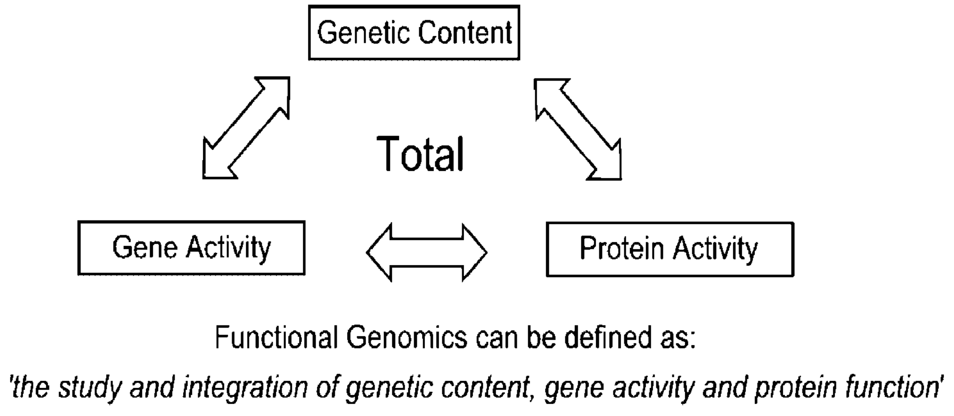

**Figure 2.** Definition of functional genomics to identify non-specific binding during the validation process. A failure rate of up to 90% during validation can occur, and this has driven the development of advanced HTS proteomic methodologies to overcome this bottleneck.

A new approach from Adaptive Screening (**http:-//www.adaptive-screening.com**) termed the Surrogate Proteome$^{TM}$, was introduced as an HTS platform technology for detecting protein–ligand binding signatures during drug discovery and development. This involves the construction of high-density arrays of fluorescently labelled protein scaffolds containing a spectrum of binding sites reactive to many drug compounds. The labelled protein scaffolds act as monitors for the binding of specific ligands, which are detected using proprietary low-light readers. The aim is to create diversity within protein-binding space. The binding signature of a ligand is monitored against known standards and its specificity can be determined. The 'ASETM' bioinformatics platform is used to provide databasing, LIMS and predictive decision-making functions with the system.

### Conclusions

Peter Ghazal (Director, SCGTI, Edinburgh, UK) concluded the meeting by highlighting the opportunities for the new methods and systems under discussion during the meeting to advance our understanding of biological systems. By definition, the emerging field of post-genomics embraces a fully integrative approach to mapping the near totality of DNA content with gene and protein activities (Figure 2). Bioarray technology is a fast-maturing science that will continue to deliver highly parallel data of an increasingly specific nature. This will impact not only academic biomedical research but also the pharmaceutical, biotechnology and healthcare sectors. Of critical importance will be the continued integration of bioinformatics and databasing. Opportunities lie ahead to generate sophisticated HTS methods to explore new areas. The potential and possibilities for bioarray and microfluidic applications seem almost limitless. Much work and interdisciplinary fusion will need to take place to realise this enormous potential. This meeting was valuable not only to reveal these concepts, but also to discuss future challenges and potentials. The meeting was well received and considered a success by the participants. Particularly appreciated was the diversity and quality of the presentations.

### Acknowledgements

The organisers would like to thank The Royal Society of Edinburgh and the Wellcome Trust for initiating and supporting this series of workshops. They would also like to thank additional sponsors, including: Scottish Higher Education Funding Council; Scottish Enterprise Edinburgh and Lothian; Engineering and Physical Sciences Research Council; MWG Biotech; Perkin Elmer; and Invitrogen. Grateful thanks are also due to Sheena Clark, Marilyn Horne and the members of SCGTI, Edinburgh, for their help in organizing this event.

Copyright © 2002 John Wiley & Sons, Ltd. 

